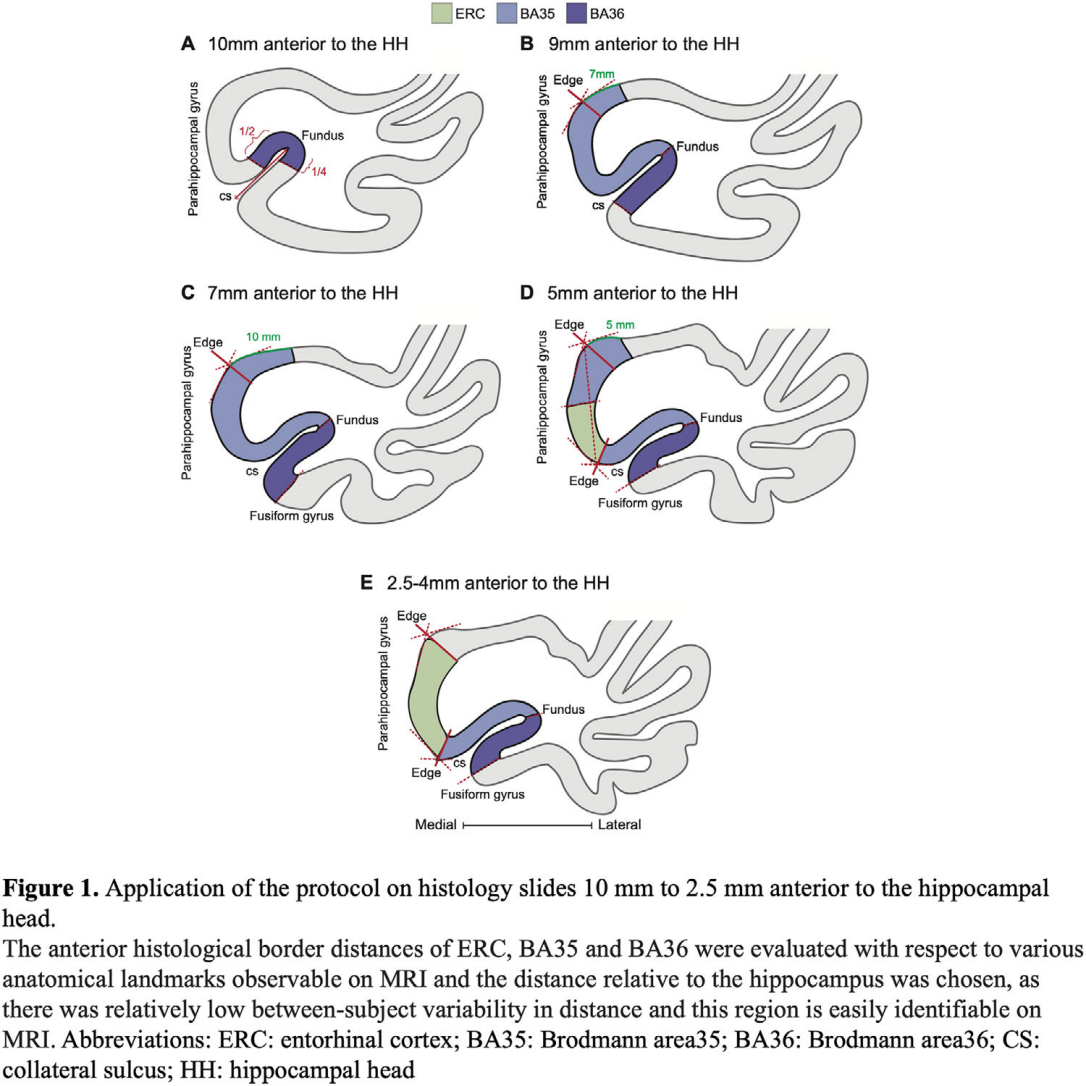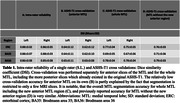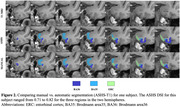# Developing an anatomically valid segmentation protocol for anterior regions of the medial temporal lobe

**DOI:** 10.1002/alz.095375

**Published:** 2025-01-09

**Authors:** Niyousha Sadeghpour, Sydney A Lim, Anika Wuestefeld, Amanda E Denning, Ranjit Ittyerah, Winifred Trotman, Eunice Chung, Shokufeh Sadaghiani, Karthik Prabhakaran, Madigan Bedard, Daniel T Ohm, Emilio Artacho‐Perula, Maria Mercedes Iniguez de Onzono Martin, Monica Munoz, Francisco Javier Molina Romero, José Carlos Delgado González, Maria del Mar Arroyo Jimenez, Maria del Pilar Marcos Rabal, Sandra Cebada Sanchez, Carlos de la Rosa Prieto, Ricardo Insausti, Corey T McMillan, Murray Grossman, Eddie B Lee, John A. Detre, John Q Trojanowski, M. Dylan Tisdall, David J Irwin, David A Wolk, Paul A. Yushkevich, Laura E.M. Wisse

**Affiliations:** ^1^ University of Pennsylvania, Philadelphia, PA USA; ^2^ Clinical Memory Research Unit, Lund University, Lund Sweden; ^3^ Department of Neurology, University of Pennsylvania, Philadelphia, PA USA; ^4^ Penn Frontotemporal Degeneration Center, Department of Neurology, Perelman School of Medicine, University of Pennsylvania, Philadelphia, PA USA; ^5^ University of Castilla‐La Mancha, Albacete Spain; ^6^ University of Castilla‐La Mancha ‐ Albacete Campus: Universidad de Castilla‐La Mancha ‐ Campus de Albacete, Albacete Spain; ^7^ Center for Neurodegenerative Disease Research, Perelman School of Medicine, University of Pennsylvania, Philadelphia, PA USA; ^8^ Department of Pathology and Laboratory Medicine, Philadelphia, PA USA; ^9^ Digital Neuropathology Laboratory, University of Pennsylvania, Philadelphia, PA USA

## Abstract

**Background:**

The anterior portion of the MTL is one of the first regions targeted by pathology in sporadic Alzheimer’s disease (AD) indicating the potential for imaging metrics from this region to serve as valuable imaging biomarkers. However, most existing automated approaches for MTL segmentation do not incorporate anterior MTL subregions, and the few that do fail to account for its complex anatomical variability. Leveraging a unique postmortem dataset consisting of histology and structural MRI scans we aimed to develop an anatomically valid segmentation protocol for anterior entorhinal cortex (ERC), Brodmann Area (BA) 35, and BA36 and apply it for automated MTL segmentation of *in vivo* 3 tesla (T) MRI.

**Method:**

We included 20 cases between 61 to 97 years of age (50% females) with and without neurodegenerative diseases (11 vs. 9 cases) to ensure broad generalizability of the developed protocol. Postmortem digitized MTL Nissl‐stained coronal histology serial sections from these cases were registered to same‐subject 0.2×0.2×0.2‐mm^3^ 9.4T postmortem MRI and annotated by an expert neuroanatomist. To develop the segmentation protocol, we determined the location of the histological borders of interest in relation to anatomical landmarks observable on *in vivo* MRI. The protocol was first applied manually to 29 3T *in vivo* MRI scans and then used to train an automatic segmentation method T1‐ASHS (Automatic Segmentation of Hippocampal Subfields). Intra‐rater reliability of a manual rater and five‐fold cross‐validation accuracy of T1‐ASHS were assessed with the Dice Similarity Index (DSI).

**Result:**

Segmentation rules for the borders of ERC, BA35 and BA36 based on systematic analysis of inter‐landmark distances on histological sections are shown in Figure 1. Intra‐rater reliability for the manual rater applying these rules to 15 *in vivo* 3T MRI scans was high (Table‐1; Figure‐2). Comparing manual segmentations with the automated ones generated by T1‐ASHS showed moderate reliability, reflecting the challenging anatomy of this region. However, segmentation accuracy for the whole MTL including the newly added region was comparable to the previously reported accuracy for MTL without this region (Table‐1).

**Conclusion:**

Future work will examine trhe utility of morphometric measures of anterior MTL regions enabled by this protocol for early AD.